# Dislocation after Posterior Stabilized Primary Total Knee Replacement: A Rare Complication in Four Cases

**DOI:** 10.1155/2021/9935401

**Published:** 2021-10-13

**Authors:** W. Spierenburg, E. L. A. R. Mutsaerts, J. J. A. M. van Raay

**Affiliations:** ^1^Department of Orthopaedic Surgery, Martini Hospital, Groningen, Netherlands; ^2^Department of Orthopaedic Surgery, OLVG Hospital, Amsterdam, Netherlands

## Abstract

**Introduction:**

Dislocation of a total knee arthroplasty is a rare complication that has rarely been described, while the total knee arthroplasty is frequently performed. From literature, we know patient-related factors, like obesity, neuropsychiatric disease, and severe valgus or varus deformity, are associated with higher risk of dislocation. We show our cases for awareness of the risk factors for surgeons. *Case Presentations*. We present four patients with a dislocation after a total knee arthroplasty. We compare these case reports with previous literature and show the most important risk factors for these dislocations. In our cases, three of them suffered from obesity, which possibly has contributed to the dislocation. Three patients did have instability which emphasizes the importance of ligament balancing while performing a total knee replacement. In all cases, an exchange of the polyethylene liner was performed.

**Conclusion:**

Implant-related factors and surgical technique as well as patient-related factors can contribute to this uncommon complication. Obesity, neuropsychiatric disorders, and a severe valgus or varus deformity are important patient-related risk factors. Our cases show these risk factors too. Some of these risk factors were encountered as well as other comorbidity factors. Such risk factors must be taken into consideration when deciding whether to perform a total knee arthroplasty. This stresses the importance of patient education and shared decision-making before performing a total knee replacement.

## 1. Introduction

Total knee arthroplasty (TKA) is one of the most commonly performed orthopaedic procedures [1]. Complete dislocation of a total knee prosthesis is a rare complication. The prevalence of knee dislocation following TKA ranges from 0.15 to 0.5% in the newer designs which incorporate changes in the height of the tibial polyethylene post [2]. Femorotibial dislocation after total knee arthroplasty has been described with fixed-bearing, mobile-bearing, cruciate-retaining (CR), and posterior-stabilized (PS) designs, although the designs that retain the posterior cruciate ligament (PCL) are the ones most commonly involved with this complication [2–6].

Causes of total knee arthroplasty dislocation can be classified into patient-related factors, surgeon-related technique, and implant designs. Patient-related factors reported from literature are comorbidity—mainly obesity, neuropsychiatric disorders, and severe preoperative deformity (varus/valgus > 10°) [7]. Surgeon-related factors are mostly linked to certain technical errors: wrong ligament balance in flexion and extension [3, 8], especially with residual laxity in flexion; excessive soft-tissue release [9]; and malalignment, including tibial implant malpositioning, specifically in internal rotation [10]. A deficient extensor mechanism can be another cause for gross instability after total knee replacement. Patient-related aspects and erroneous surgical choices are the most significant risk factors for dislocation [7].

We present four patients with a femorotibial dislocation after a Genesis total knee arthroplasty, posterior-stabilized, and disengagement of the polyethylene insert. Dissociation of the polyethylene insert from the tibial baseplate in PS Genesis II TKA has been previously described [11, 12]. We present the contributing factors in our cases for this rare complication together with the treatment options.

## 2. Case Presentations

### 2.1. Case 1

A 71-year-old woman came to the emergency room of our hospital because of intense pain in her right knee after getting up from a chair. She had a history of lung emphysema, diabetes, and obesity and underwent a total knee replacement in 2014 (Genesis II, posterior-stabilized) for osteoarthritis on the right side with a normal leg alignment. Patient characteristics are mentioned in Table 1.

PS: posterior-stabilized; M: male; F: female; BMI: body mass index; COPD: chronic obstructive pulmonary disease; CVA: cerebrovascular accident.

**Figure 1:**
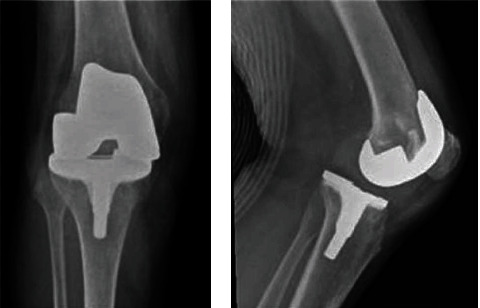
(a) Anteroposterior radiograph of the knee from case 1 at presentation in the emergency room. (b) Lateral radiograph of the knee from case 1 at presentation in the emergency room.

Her right knee was flexed 45° and she could extend maximally up to 25°. The knee was not swollen and showed no redness. ESR and CRP were within normal limits. Conventional X-rays of the right knee indicated anterior to posterior tibiofemoral dislocation of the Genesis PS knee prosthesis (Figures 1(a) and 1(b)).

At the emergency room, we tried to reduce the knee prosthesis by giving traction on the tibia and pulling the tibia anteriorly in the hyperflexed knee. This procedure was not successful. On the operation room, we used the medial parapatellar approach to approach the knee. In all four cases during revision surgery, we used this approach, the same as the initial approach. In all knees, we used the anterior reference technique to balance the knee.

During surgery, disengagement of the liner was seen, and the liner was removed. The liner showed no wear or sublaminar cracks. A new thicker liner was placed, and the knee was stable in flexion and in extension. The patient was confident, and the postoperative course was uneventful.

### 2.2. Case 2

**Figure 2:**
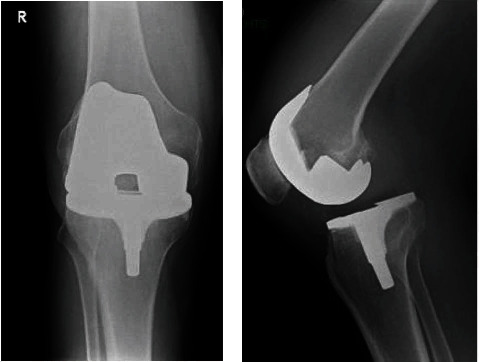
(a) Anteroposterior radiograph of case 2 at presentation in the emergency room. (b) Lateral radiograph of case 2 at presentation in the emergency room.

A 63-year-old male with a history of an ischaemic cerebrovascular incident in 1987 with a hemiparesis on the left side was referred to the emergency room. In 2016, he underwent a replacement of a PS Genesis 2 total knee prosthesis for osteoarthritis on his right knee, which was a valgus knee. He only made transfers between bed and wheelchair at home. While sitting in his wheelchair, he suddenly felt intense pain in his right knee without a history of trauma. He was unable to flex or extend the knee. X-rays showed a tibiofemoral dislocation of the knee prosthesis (Figures 2(a) and 2(b)). Reduction at the emergency room was not successful. We performed surgery using again the medial parapatellar approach. The liner was unaffected but showed posterolateral instability in flexion and extension. The knee stabilized postoperatively after exchanging the liner for a thicker one because of collateral laxity in flexion and extension. After the operation, the patient was able to make painless transfers, just like he could before.

### 2.3. Case 3

**Figure 3:**
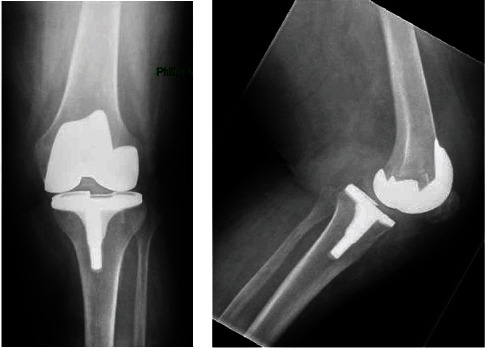
(a) Anteroposterior radiograph of case 3 at presentation in the emergency room. (b) Lateral radiograph of case 3 at presentation in the emergency room.

An 81-year-old woman underwent a total knee prosthesis with a patellar component because of medial and patellofemoral osteoarthritis of the left knee with a normal knee alignment. She suffers from obesity, diabetes, atrial fibrillation, hypertension, depression, and cardiac arrhythmia. After rehabilitation, the patient was confident about her knee and walked without problems. Eight months later, while going to bed, she hyperflexed her left knee and felt intense pain afterwards. Bending the knee was impossible and she was referred to the outpatient clinic. X-rays showed a tibiofemoral dislocation (Figures 3(a) and 3(b)). A closed reduction was performed, but a persistent disengagement of the liner was observed afterwards. In surgery, using again the medial parapatellar approach, the liner dissociated from the tibial tray during hyperflexion of the knee and internal rotation of the lower leg. An internal rotation position of the tibial component was observed. A new thicker liner was placed, and the knee stabilized in flexion and extension.

### 2.4. Case 4

**Figure 4:**
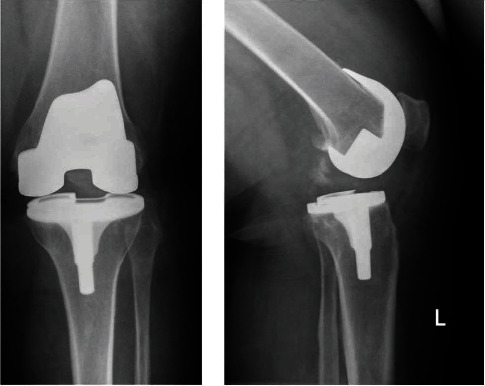
(a) Anteroposterior radiograph of case 4 at presentation in the emergency room. (b) Lateral radiograph of case 4 at presentation in the emergency room.

A 46-year-old woman underwent a total knee replacement (Genesis type PS) in 2016 for end-stage varus osteoarthritis of her left knee. She has a history of lung embolism, depression, Sjögren's syndrome, COPD, and obesity (BMI 55.6). After the rehabilitation period, the patient complained about instability. Upon examination, her medial collateral ligament appeared too loose. The patient agreed with exchanging the liner to a thicker one. While waiting for the operation, she injured her left knee walking. She felt intense pain and was unable to walk. Her knee was swollen, and she was unable to bend her knee further than 40° or extend further than 20°. X-rays showed a dislocation of the insert of the knee prosthesis (Figures 4(a) and 4(b)). Closed reduction in the operation room was successful. One month later, the liner was revised to a thicker one because of a persistent unstable feeling using again the medial parapatellar approach. The knee showed a laxity medial in flexion and extension. Laxity disappeared after placing a thicker liner. After this procedure, the patient is very confident, and the knee was stable in flexion and extension. Three years after the dislocation, she decided to undergo a total knee replacement for her right knee, and her left knee remained uneventful.

## 3. Discussion

All of the patients had a Genesis II total knee arthroplasty. The posterior-stabilized design has a femoral cam and a tibial post to produce femoral rollback, thereby increasing the potential range of flexion. Still, there is a critical point beyond which an implant design allowing increasing flexion range would compromise knee stability. The design is not a factor in these cases, because this complication occurs in all different designs, especially in the ones who retain the posterior cruciate ligament [2–5]. Even more, Jeffcote et al. and Nicholls et al. describe that except for the initial choice for the constraint, for example, a cruciate retaining design with an insufficient cruciate ligament, the design is not a factor anymore for dislocation, especially not in the newer posterior stabilized design [13, 14].

Tibiofemoral dislocation after total knee replacement with disengagement of the polyethylene liner is a rare complication [4], first reported by Insall et al. in 1979 after total condylar knee replacement in four patients out of a series of 220 patients [15]. In literature, a prevalence of knee dislocation following TKA ranges from 0.15 to 0.5% [2]. The dislocations from our case series took place in two hospitals. One dislocation took place in a hospital where around 400 total knee replacements were performed per year. In the other hospital, 450 total knee replacements were performed per year. We noticed the complication four times over five years in a total of 950 primary total knee replacements. This makes a percentage of 0.08% in our population. This is a lower percentage than in literature [2], possibly because the percentages from earlier research are calculated a long time ago, twenty years ago, and meanwhile, implants, surgical technique, and doctor's indications have been improved.

The patient of the first case had a patient-related risk factor, obesity, described by Rouquette et al. [7]. They published a systematic review on total knee dislocation in primary total knees and identified influencing factors [7]. Causes of dislocation were divided into patient-related factors, surgeon-related factors, and erroneous initial choice of implant. The most important patient-related factors were obesity (39.2%), neuropsychiatric disorders (10.2%), and severe preoperative deformity (varus/valgus > 10°) [7]. Overall comorbidities were the main factor (65.2%), followed by intraoperative iatrogenic lesions (collateral ligament lesions, extensor system destabilization, or implant malpositioning, at 60.9%).

The theory of obesity leading to more dislocations is simply explained by increased mechanical stress on the prosthesis [16]. Neuropsychiatric disorders leading to dislocation are explained by peripheral neuromuscular disorder underlying desynchronization of joint agonist/antagonist muscles [17].

The patient in case 1 was not satisfied with her total knee. This can be due to different reasons. One possible cause is flexion instability: if it increases over time, it would explain the dislocation. Instability after total knee arthroplasty can be classified as mediolateral, anteroposterior, rotational, or flexion instability. Patients with a flexion instability after total knee arthroplasty mostly report vague pain and swelling after activity. There is laxity in the varus and valgus strain in flexion as well as an anterior subluxation of the femur on the tibia in flexion rather than a rollback. The forward rolling of the femoral component can be seen on the flexion lateral X-ray.

It is also doubtful whether manual reduction could have a successful outcome at the emergency room in this patient, who was in a lot of pain. Repositioning of the dislocation can take place conservatively by manual reduction or operatively. Manual reduction could be difficult, given the need for muscle relaxation, and this can only be achieved using sufficient analgesics. Additionally, there can be potential vascular (popliteal artery) and ligamental structures like the PCL (in posterior cruciate retaining designs), or the collateral ligaments can be affected too. At the same time, conservative treatment entails a risk of recurrence [7], so operative treatment is mostly recommended.

Case 2 shows a knee prosthesis placed in a patient who does not really walk and therefore had poor muscle quality due to a past CVA. The patient additionally had a preexistent valgus knee, which can cause posterolateral laxity because of perioperative releases while placing the knee prosthesis. These two “problems” could have caused a laxity which may have been the reason for the dislocation.

The patient of case 3 had multiple comorbidities plus obesity. Some possible surgeon-related factors may have contributed to dislocation of the liner. While revising the liner, an abnormal position of the tibial implant and flexion instability was seen. The tibial implant was placed in internal rotation and the liner dislocated in flexion. This can cause rotational instability, as described in earlier cases due to ligamental imbalance or component malpositioning [4]. Hence, in this case, a combination of factors, patient- and surgeon-related, would have caused the dislocation.

The fourth case shows a combination of complicated patient factors like multiple comorbidities and obesity in combination with varus osteoarthritis at a very young age. The possible complications were thoroughly considered before the operation, but the chances of complications were a lot higher in this patient. She suffers from obesity, a neuropsychiatric disorder (depression), two main complicated factors described by Rouquette, and laxity of her medial collateral ligament preoperatively because of her varus osteoarthritis.

Strikingly, all four patients were in poor condition, in accordance with the literature [7]. The fourth case shows the importance of knee stability; hence, optimal soft-tissue tension balance should be required for surgery. All of our cases were revised by replacing the liner with a thicker one, while Rouquette et al. describes a total revision rate of 80% [7], although, after liner replacement, all our four knees showed collateral stability in flexion, midflexion, and extension.

Different treatment modalities can prevent new dislocations. The best treatment can depend on the cause. In a cruciate-retaining design, one can simply increase the height of the insert, but this procedure has been associated with high failure rates of 35.7% [18]. Replacing a cruciate-retaining design with a posterior-stabilized design is a treatment that prevents dislocation with good results and is the gold standard [19]. With severe instability, there can be component malpositioning or frequent dislocations. In such cases, complete revision or an intercondylar constrained design is necessary. A hinged or constrained prosthesis has tibial and femoral components that are linked together with a hinged mechanism, which can be useful in extreme unstable knees. Perhaps we should have considered total revision in our cases more strongly, despite the obtained collateral stability after liner replacement.

## 4. Conclusion

These cases illustrate that dislocation of a posterior-stabilized total knee arthroplasty can occur. Our cases show the influence of patient-related factors like obesity, neuropsychiatric diseases, other comorbidities, and preoperative alignment.

## Figures and Tables

**Table 1:** Patient characteristics.

	M/F	BMI	Age at primary TKA	Time between TKA and dislocation	Design	Comorbidities	Knee alignment at time of initial knee replacement	How was the problem solved?
Case 1	F	34.9	69	2 years	PS	Obesity, lung emphysema, diabetes	Normal	Replace the liner with a thicker one
Case 2	M	23.1	61	2 years	PS	CVA with hemiparesis	Valgus alignment	Replace the liner with a thicker one
Case 3	F	32.6	81	8 months	PS	Obesity, diabetes, atrial fibrillation, hypertension	Normal	Replace the liner with a thicker one
Case 4	F	55.6	46	10 months	PS	Obesity, COPD, depression, lung embolism, Sjögren's syndrome	Varus alignment	Replace the liner with a thicker one

## Data Availability

We shall make data available to researchers who provide a methodologically sound proposal to achieve the aims in this proposal. They could get the individual participant data that underlie the results reported in this article. Data can be obtained by contacting the corresponding author. Data are available beginning 3 months and ending 5 years after article publication.

## Additional Points

*Clinical Message*. Tibiofemoral dislocation is a very rare complication in a frequently performed operation, the TKA. We recommend proper selection and ensuring that the patient weighs the known and other potential risks before undergoing a total knee replacement. Furthermore, be aware of vascular and ligamental damage and consider total revision in patients with a femoral dislocation of a total knee replacement.
